# PROTOCOL: Evidence and gap map protocol: Institutional responses to child maltreatment

**DOI:** 10.1002/cl2.1039

**Published:** 2019-09-13

**Authors:** Bianca Albers, Caroline Fiennes, Aron Shlonsky, Meghan Finch, Ludvig Bjørndal, James Hennessy, Joachim Krapels, Rebecca Featherston, Robyn Mildon

**Affiliations:** ^1^ Centre for Evidence and Implementation London UK; ^2^ Giving Evidence Cornwall UK; ^3^ School of Primary and Allied Health Care Monash University Peninsula Campus Frankston Victoria Australia; ^4^ Centre for Evidence and Implementation Carlton Victoria Australia; ^5^ Porticus Amsterdam The Netherlands; ^6^ Department of Social Work School of Primary and Allied Health Care, Faculty of Medicine Nursing & Health Sciences Monash University Peninsula Campus Frankston Victoria Australia

## 1 BACKGROUND

### 1.1 The problem, condition or issue

Child maltreatment, that is, ‘various types of violence such as sexual, emotional and physical, abuse and/or emotional and physical neglect’ (Lueger‐Schuster et al., 2018), is a widespread phenomenon affecting millions of children, adults and communities around the globe.

Measuring the prevalence of child maltreatment in institutional contexts is challenging, and these data are not readily available. Moreover, studies do not generally deal solely with maltreatment occurring specifically in these settings, and disentangling the effects of maltreatment occurring in institutional settings versus other maltreatment settings is not routine. That said, the overall rates of any type of child maltreatment may provide some indication, and these have recently been estimated to be between 3 per 1,000 children for physical and emotional abuse and 4 per 1,000 children for sexual abuse (Stoltenborgh, Bakermans‐Kranenburg, Alink & van IJzendoorn, 2015). The World report on violence and health launched in 2002 reported a prevalence for abandonment and neglect at 21.9% in Kenya and 12–19% of physical neglect and abandonment in Canada (Krug, 2002). A series of meta‐analyses estimated an overall prevalence rate of 16.3% for physical neglect and 18.4% for emotional neglect (Stoltenborgh et al., 2015). However, prevalence rates are sensitive to a number of factors. There are both geographical and gender differences. Rates also vary depending on whether incidences of maltreatment are self‐reported or based on informants (Greger, Myhre, Lydersen & Jozefiak, 2015; Moody, Cannings‐John, Hood, Kemp & Robling, 2018), and can vary with the identity of the perpetrator/s. How widely or narrowly different subtypes of maltreatment are defined and operationalised in studies or how many items are used to measure prevalence, can also impact on rate estimates. They should therefore be interpreted with caution.

Even less is known about the prevalence of various forms of child maltreatment taking place within institutional settings such as kindergartens and schools, religious institutions, out‐of‐home care and other comparable contexts in which children spend their time (Blakemore, Herbert, Arney & Parkinson, 2017; Proeve, Malvaso & DelFabbro, 2016). Maltreatment in these contexts, can encompass adults abusing children, children abusing other children, institutions enabling child maltreatment and child characteristics enhancing their vulnerability to maltreatment. The dearth of research is due to the comparably young age of institutional child maltreatment as a field of empirical research (Timmerman & Schreuder, 2014), in which the focus has been on sexual abuse within especially religious and out‐of‐home care institutions, whereas other types of maltreatment and settings have been less examined (Proeve et al., 2016).

Recent studies conducted in Germany (Allroggen, Ohlert, Rau & Fegert, 2018) and Norway (Greger et al., 2015) confirm that children placed in institutional care are at significantly higher risk of experiencing maltreatment but less is known about maltreatment taking place in areas such as sports and exercise (Bjørnseth & Szabo, 2018).

However, it is clear that child maltreatment taking place in these settings affects the lives of both victims, their families and their communities—at times for generations. Child maltreatment has a negative impact on the physical, mental, spiritual, educational and economic wellbeing of those experiencing and surviving it—both in the short term and in the form of long‐term consequences that reduce the quality of life during adulthood (Lueger‐Schuster et al., 2018).

In recent years, child maltreatment occurring in institutional settings has received substantial attention both at the policy level, amongst practitioners and service agencies working with children in different capacities and roles, and as part of the public debate.

The shift in attention and prioritisation of child maltreatment as a key concern of society is reflected in a broad range of official inquiries and key reports conducted in recent years in especially high‐income countries—of which the following is a selected sample:

- Law Commission of Canada (2012). Restoring Dignity—Responding to Child Abuse in Canadian Institutions.
- The Scottish Government (2012). Review of Child Neglect in Scotland.
- New Zealand House of Representatives (2014). Inquiry into Improving Child Health Outcomes and preventing child abuse with a focus from preconception until three years of age.
- Royal Commission into Institutional Responses to Child Sexual Abuse (Australia, 2014–2017).
- Northern Ireland Historical Institutional Abuse Inquiry 1922–1995 (2017).
- Pennsylvania Attorney General (2018). Pennsylvania Diocese Victims Report.
- Unabhängige Kommission zur Aufarbeitung sexuellen Kindesmissbrauchs (Germany, 2016–2023).
- Indepedent Inquiry Child Sexual Abuse (U.K., 2019).
- Sexual Abuse of Children in Custodial Institutions (U.K., 2009–2017).

These inquiries have led to the production of many research reports—among them a number of rapid or full systematic reviews examining the impact of institutional child maltreatment (Blakemore et al., 2017), how it can be prevented (South, Shlonsky & Mildon, 2015, 2014; Pitts, 2015), victims supported (Shlonsky, Albers & Paterson, 2017), and suitable responses be implemented and maintained over time (Parenting Research Centre, 2015; Albers & Mildon, 2016).

However, this and other evidence on the effectiveness of interventions aimed at identifying, preventing or responding to institutional child maltreatment is spread across multiple sources and often exists in the form of academic or grey literature that can be difficult to access for institutions that wish to improve their practices and services in this area.

Therefore, there is still considerable confusion amongst sector stakeholders about what evidence exists for safeguarding interventions developed for use in institutional settings. The objective of the evidence and gap map (EGM) described in this protocol is to reduce this confusion and to provide a ‘go to’ knowledge base for stakeholders wanting to access high‐quality evidence on interventions addressing institutional child maltreatment.

### 1.2 Scope of the EGM

The guiding research question for this evidence and gap map is:

***What is the prevalence of evidence on the effectiveness of interventions that***—***within institutional settings***—***are aiming to***

- Prevent the occurrence of maltreatment of children (including preventing peer to peer abuse)
- Prevent the recurrence of maltreatment of children (preventing offenders from re‐offending)
- Reduce harm to the health and wellbeing of children exposed to child maltreatment
- Enhance the disclosure of child maltreatment; and
- Improve organisational practice and standards for addressing issues related to child maltreatment.

Guided by this research question, the EGM will be structured into interventions aimed at institutional child maltreatment identification/disclosure, prevention, treatment and other responses (vertical structure). The EGM's horizontal structure will be formed by outcomes that relate to the institutional setting, the child's physical, mental, spiritual, educational and economic wellbeing, and the perpetrators of child maltreatment. These dimensions of the EGM are outlined in greater detail below—under ‘EGM Framework’.

The EGM will contain effectiveness studies of different designs, including overviews of systematic reviews, systematic reviews, (cluster) randomised controlled trials and studies using quasi‐experimental designs. It will be a global EGM covering low‐, middle‐ and high‐income countries. In the following, these and other characteristics are described in greater detail.

### 1.3 Conceptual framework of the EGM

Child maltreatment in institutional settings is a complex problem consisting of four potential factors influencing the occurrence of child maltreatment (Royal Commission into Institutional Responses to Child Sexual Abuse, 2017):

- Adults abusing children,
- Children abusing other children,
- Institutions enabling child maltreatment, and
- Child characteristics enhancing their vulnerability to maltreatment

In targeting child maltreatment, interventions may have a different scope and be aimed at:

- ***Preventing occurrence and reoccurrence of child maltreatment.*** This may be based on either
  – Universal services available to an entire target population and aimed at promoting positive behaviours and functioning and/or at decreasing risk factors and the likelihood of problems and challenges in a person's life.
  – Targeted services available to selected members of a target population who are at risk of developing or experiencing particular problems—with the intervention aimed at reducing these risks.
- ***Disclosing child maltreatment.*** A key factor in ending, responding to and treating the consequences of child maltreatment is its disclosure—especially in cases of child sexual abuse (Paine & Hansen, 2002; Lemaigre, Taylor & Gittoes, 2017). Recent inquiries have documented the substantial barriers existing in institutional settings to facilitate such disclosure (Royal Commission into Institutional Responses to Child Sexual Abuse, https://www.childabuseroyalcommission.gov.au/; Lemaigre et al., 2017), pointing to the importance of including disclosure interventions in this EGM.
- ***Responding to the occurrence of child maltreatment.*** Institutions have strong legal and ethical obligations to respond appropriately when child maltreatment has been detected or disclosed. This includes reporting the maltreatment, supporting the victim and/or family, working with child protection agencies and providing training and crisis support to staff.
- ***Treating the consequences of child maltreatment***. Providing services or referring children and families to agencies that provide therapeutic care for one or more of the many known problems associated with experiencing child abuse and neglect (e.g., PTSD).

Based on this understanding, the EGM will cover studies examining interventions aimed at preventing occurrence and reoccurrence of child maltreatment, disclosing child maltreatment, responding to the occurrence of child maltreatment and/or treating its consequences.

These interventions may be placed at all levels of the service spectrum and target either children, child offenders, perpetrators or the institutional setting.

Different organisational factors have been identified that purportedly increase or decrease the likelihood of institutional child abuse (Royal Commission into Institutional Responses to Child Sexual Abuse, 2017), including institutional:

- Cultural factors (e.g., leadership, organisational culture),
- Operational factors (e.g., governance, day‐to‐day work routines and practices), and
- Environmental factors (e.g., physical spaces).

Studies examining interventions addressing any of these organisational factors will therefore be included in this EGM.

A more detailed outline of how this overarching framework will be operationalised in developing the full EGM is presented in Section 3 of this protocol.

### 1.4 Why it is important to develop the EGM

Given the lack of a ‘go to’ global knowledge base presenting high quality evidence on the effectiveness of safeguarding (actions taken to protect vulnerable groups from harm; Cambridge University Press, 2019) interventions tested in institutional settings, the production of this EGM is highly overdue.

The knowledge it will generate will have the potential to support:

- The identification of areas with potentially sufficient studies to conduct a meta‐analysis within a systematic review ‐ if none currently exists.
- Service providers (institutions) in identifying potentially effective interventions and/or key characteristics of potentially effective interventions. This knowledge can be used to inform the selection or design of safeguarding interventions to be applied locally.
- Funders and policymakers to inform funding and/or policy decisions related to the safeguarding of children in institutional settings. These can be decisions about the selection of potentially effective interventions or the funding of research—for example, research that can fill existing gaps in the knowledge base.
- Research organisations in assessing the current evidence on child maltreatment in institutional settings. This knowledge can inform the development of research agendas and priorities.

### 1.5 Existing EGMs and/or relevant systematic reviews

To our knowledge, there are only three other evidence and gap maps that—in different ways—relate to issues of child maltreatment—all of which are registered with the Campbell Collaboration:

1. Kornør, John, Axelsdottir, Biedilæ and Albers (2018) is in development still. It will focus on
  a. Child maltreatment
  b. Children aged prenatal‐12 years
  c. Studies conducted in high‐income countries only.

The focus of this EGM is child abuse and neglect in general, with a focus on clinical interventions and hence does not keep a focus on institutional settings where the evidence on the effectiveness of non‐clinical interventions aimed at enhancing, for example, organisational standards or disclosure rates, is of particular interest.

2. Saran, Albright, Adona & White (2018) has been developed in full and is available in the public domain. It focuses on
  a. Child welfare
  b. Children aged under 18
  c. Studies conducted in low‐ and middle‐income countries (LMICs) only.

This EGM includes 302 systematic reviews on a broad range of child welfare interventions and outcomes with a focus on evidence relating to LMICs. Interventions addressing child abuse are a minor element of this EGM, no particular focus on institutional settings is described, and studies conducted in high‐income countries are not included.

3. Pundir, Saran, White, Adona, and Subrahmanian (2019) is in development. It will focus on:
  a. Violence against children
  b. Children under 18 years
  c. Studies conducted in LMICs only

This EGM will provide an overview of the evidence on the effectiveness of interventions aimed at reducing violence against children in LMICs. As for Saran et al. (2018) described above, no particular focus on institutional settings is described, and studies conducted in high‐income countries are not included

Taken together, this means that the EGM described here will be a genuine and much needed contribution to the evidence base on child maltreatment because it:

- focuses on *institutional settings*—which are not the key focus of any of the other evidence and gap maps and therefore may be at risk of ‘disappearing’ in large amounts of other evidence regarding child maltreatment occurring in other contexts; and
- keeps a global focus and combines the evidence existing for high‐ with that from low‐ and middle‐income countries.

As such, this EGM will be a substantial resource for a broad range of stakeholders operating in child health, welfare and education sectors. This includes kindergartens, schools, charities, churches, sports clubs, scouting associations, out‐of‐home‐care providers and the many other organisations that have children in their care on a daily basis—and their funders.

## 2 OBJECTIVES

The objectives of this EGM are twofold. It will

a) Provide a structured and accessible collection of existing evidence from finalised and ongoing overviews of systematic reviews, systematic reviews and effectiveness studies of institutional responses to child maltreatment ‐ for those who work to fund, develop, implement, and evaluate interventions aimed at ensuring children's safety in institutional settings.
b) Identify gaps in the available evidence on institutional responses to child maltreatment ‐ thereby helping to inform the research agendas of funders and other organisations.

## 3 METHODOLOGY

### 3.1 Defining evidence and gap maps

Mapping the evidence in an existing area is a relatively new approach that has been used since the early 2000s (Saran & White, 2018). EGMs are ‘evidence collections’ (Snilstveit, Vojtkova, Bhavsar & Gaarder, 2013, pp. 3) that provide a visual overview of the availability of evidence for a particular sector—in this case, institutional responses to child maltreatment. They belong to a group of evidence synthesis products that aim to ‘configure information’ (Littell, 2018, pp.10). They help to consolidate what we know and do not know about studies that evaluate the effectiveness of interventions in a given area ‐ by mapping out existing and ongoing systematic reviews and impact evaluations in this field, and by providing a graphical display of areas with strong, weak or non‐existent evidence on the effect of such interventions.

Studies included in an EGM are identified through a comprehensive search of published and unpublished literature, which targets both completed and ongoing studies—the latter to help identify research in development, which might help fill existing evidence gaps.

The methods for conducting EGMs draw on the principles and methodologies adopted in existing evidence mapping and synthesis products. Typically, six steps are taken when conducting an EGM:

*Step 1. Defining scope*

The first step in producing an EGM is to set the scope by developing a framework, which represents the universe of interventions and outcomes in the field to be covered. The rows of the framework cover all interventions relevant to the sector covered, while columns include all relevant outcomes, from intermediate outcomes to final outcomes.

*Step 2. Setting study inclusion criteria*

As part of this step, the types of evidence to be included in the EGM are determined. EGMs often rely on two types of studies: (a) systematic reviews that critically appraise and synthesise all available evidence in a particular area; (b) and impact evaluations that rigorously test effectiveness using rigorous experimental and quasi‐experimental designs.

*Step 3. Searching for studies and assessing inclusion*

Next, a strategy for populating the EGM framework with studies meeting the study inclusion criteria is developed. The methods for doing so draw on methods of systematic searching commonly used for systematic reviews and overviews of reviews.

*Step 4. Coding and critical appraisal*

This step involves the systematic coding and extraction of data using a structured format. Studies are coded according to relevant intervention and outcome categories. The quality of the included systematic reviews and impact evaluations is also appraised.

Depending on the purpose of the EGM and the needs of stakeholders, other coding categories may also be relevant, including, for example, geographical scope of the evidence, demographic characteristics of target populations, study settings etc.

*Step 5. Producing user‐friendly summaries, presentations and analysis*

A key feature of an EGM is to provide direct access to user‐friendly summaries. The method for this—and the final functionality of the map—will often depend on the resources available to produce the EGM.

*Step 6. Further disseminating knowledge derived from the EGM*

Finally, the map itself, together with information about its key findings and use, will be disseminated to its key users and other stakeholders, for example, through presentations, webinars, research briefs and other means.

In the following, we outline how these steps will be taken for the EGM on child maltreatment in institutional settings.

### 3.2 EGM framework

#### 3.2.1 Population

This EGM will focus on the universe of interventions and outcomes for children

- aged under 18 years at the point of baseline measurement,
- living in and/or engaging in activities in institutional settings.

Whilst children are the key target population, study participants may still be adults in that this EGM aims to include evidence on interventions that improve the professional practice of staff and organisational standards of agencies providing services to children and families.

This EGM will be based on the framework visually outlined below.

In the following we will first define intervention categories, followed by outcome categories.

#### 3.2.2 Intervention categories

This EGM will focus on four different intervention categories. Within each of these, the subcategories remain the same, as interventions are classified by their primary target—the child (victim), the offender/perpetrator or the institutional/organisational context in which child maltreatment takes place.

The following table provides definitions for each intervention category and examples of how these may relate to each subcategory of key targets:

**Table:** 

Intervention category	Subcategory	Examples
***Prevention***	Victim	− Universal/primary interventions (e.g. Educational interventions used in school settings, maternal‐child health screening)
		− Tertiary interventions (e.g. Advocacy, social supports)
	Perpetrator	− Universal/primary interventions (e.g. Traditional or social media campaigns)
		− Targeted therapeutic interventions (e.g. CBT group therapy, education interventions)
		− Tertiary interventions (e.g. criminal justice, pre‐employment screening/criminal background checks)
	Organizational context	− Staff training / professional development (e.g. Education programs)
		− Organisational guidelines and/or practices
		− Legal/policy interventions aimed at introducing new procedures for institutions to follow (e.g. Response framework)
		− Particular institutions aimed at enhancing safeguarding practice and outcomes in institutional settings (e.g. Child Advocacy Centres)
***Disclosure***	Victim	− Universal/primary interventions (e.g. Traditional or social media campaigns, abuse helplines)
		− Targeted therapeutic interventions (e.g. Play therapy)
	Perpetrator	− Legal interventions (e.g. Mandatory reporting)
	Organizational context	− Staff training / professional development
		− Organisational guidelines and/or practices (e.g. Guidelines for reporting abuse)
		− Legal/policy interventions aimed at introducing new procedures for institutions to follow (e.g. Response framework)
		− Particular institutions aimed at enhancing safeguarding practice and outcomes in institutional settings (e.g. Child Advocacy Centres)
***Response***	Victim	− Tertiary interventions (e.g. Legal avenues for criminal redress, advocacy, social supports)
	Perpetrator	− Tertiary interventions (e.g. Criminal justice, arrest, removal of credentials)
	Organizational context	− Staff training / professional development
		− Organisational guidelines and/or practices (e.g. Response framework, perpetrator accountability)
		− Legal/policy interventions aimed at introducing new procedures for institutions to follow
		− Particular institutions aimed at enhancing safeguarding practice and outcomes in institutional settings (e.g. Child Advocacy Centres)
***Treatment***	Victim	− Targeted therapeutic interventions (e.g. Trauma‐focussed interventions)
	Perpetrator	− Targeted therapeutic interventions (e.g. CBT group therapy, behaviour change programs, narrative therapy)
	Organizational context	− Staff support (e.g. Staff counselling)

Studies in which only a subset of the interventions covered are eligible for inclusion in the map will be included, provided that the outcomes reported for these interventions are of relevance to this EGM.

#### 3.2.3 Outcome categories

This EGM will focus on six different outcome domains

**Table:** 

Outcome category	Subcategory	Examples
***Institutional safeguarding practice***	Cultural changes	− Leadership behaviour (e.g. role modelling of safeguarding behaviour)
		− Staff perceptions of the importance of safeguarding / risk‐aware / risk‐targeting behaviour
	Operational changes	− Staff recruitment procedures to enhance safeguarding practices
		− Staff training to increase knowledge and awareness
		− Implementation of child safeguarding policies
	Environmental changes	− Changes in the institution's physical environment
***Child maltreatment disclosure***	Disclosure rates	− The disclosure of different types of maltreatment through the victim, caregivers, institutional staff or others involved in the child's life
***Child maltreatment occurrence or reoccurrence (child safety)***	Neglect	− The occurrence or re‐occurrence of different types of child maltreatment within the institutional setting, for study participants – measured e.g. through self‐ or informant‐reports
	Emotional and Psychological Abuse	− Feelings of personal safety and security
	Physical Abuse	− Presence of relationships that facilitate disclosure and / or harm
	Sexual Abuse	
	Child knowledge / awareness	− Knowledge about child maltreatment and potential responses to offending behaviour
		− Risk‐aware / risk‐targeting behaviour
***Child health and wellbeing***	Physical Health & Development	− Normative standards for growth and development
		− Gross motor and fine motor skills
		− Overall health
		− BMI
		− Risk‐avoidance behaviour related to health
	Mental health	− Self‐control, emotional management and expression
		− Internalizing and externalizing behaviours
		− Trauma symptoms
		− Self‐esteem
		− Emotional intelligence
		− Self‐efficacy
		− Motivation
		− Prosocial behaviour
		− Positive outlook
		− Coping
	Social‐emotional functioning	− Social competencies and skills
		− Attachment and caregiver relationships
		− Adaptive behaviour
		− Social connections and relationships
	Cognitive functioning	− Language development
		− Pre‐academic skills (e.g., literacy / numeracy)
		− Approaches to learning
		− Problem‐solving skills
		− Academic achievement
		− School engagement / school attachment
***Adult perpetrator / child or youth offender outcomes***	Desistance	− The degree of cessation of the maltreating behaviour
	Recidivism	− The occurrence of relapse into maltreating behaviour
	Maltreatment behaviours	− Harmful coercive behaviours
		− Bullying behaviours
		− Problem Sexual Behaviour (children under 10)
		− Harmful sexual behaviour (children aged from 10 up to 18)
		− Sexually offending behaviour (children aged from 10 up to 18 receiving treatment through a juvenile justice intervention)
***Parent / caregiver outcomes***	Parent / caregiver knowledge / awareness	− Knowledge about institutional policies and practices required to safeguard children
	Parent / caregiver behaviour	− Responsiveness to lack of institutional standards

Framework: EGM on child maltreatment in institutional settings. In combining intervention with outcome categories, this EGM will utilise the following framework (see following page):

**Table:** 

*Outcomes*		*Inst. Safeguarding Practice*			*Disclosure*	*Child maltreatment occurrence or reoccurrence (child safety)*				*Child health & wellbeing outcomes*					*Perpetrator/Offender outcomes*				*Caregiver outcomes*	
*Intervention purpose/target*		Culture	Operations	Environment	Disclosure rates	Neglect	Emotional abuse	Physical abuse	Sexual abuse	Knowledge/awareness	Physical health	Mental health	Social‐emotional functioning	Cognitive functioning	Desistance	Recidivism	Recidivism	Behavioural Health	Knowledge/awareness	Behaviour
**Disclosure**	*Victim*																			
	*Perpetrator*																			
	*Org. context*																			
**Prevention**	*Victim*																			
	*Perpetrator*																			
	*Org. context*																			
**Response**	*Victim*																			
	*Perpetrator*																			
	*Org. context*																			
**Treatment**	*Victim*																			
	*Perpetrator*																			
	*Org. context*																			

#### 3.2.4 Adverse outcomes

This EGM will include any measure of adverse outcomes relating to the included interventions and outcome categories. All adverse effects described in eligible studies will be included in the synthesis. Unintended adverse effects may include a range of outcomes affecting victims, perpetrators or institutions.

Some examples of potential adverse effects may include:

- Increased lack of trust or fear of adults / institutions
- Reduced opportunities for children to interact with adults who are safe
- Negative effects of early exposure to knowledge about emotional, physical, sexual or other abuse that is not developmentally appropriate
- Reduced privacy for children as a result of safeguarding the environment
- Increased risk of retraumatisation of child or adult victim survivors of abuse
- Decrease in male role models (due to females being chosen more often than men to undertake roles that involve children)
- Reduced privacy for accused perpetrators
- Negative financial and / or reputational repercussions for institutions.

### 3.3 Criteria for including and excluding studies

#### 3.3.1 Types of study designs

This EGM is focused on studies that report on the effectiveness of child maltreatment interventions used within institutional settings and will include finalised and ongoing overviews of systematic reviews, systematic reviews and effectiveness studies. Systematic reviews and overviews of reviews will be included where they report replicable methods to synthesise and summarise available research evidence to answer a well‐defined research question. Systematic reviews with and without meta‐analyses will be included.

Given potential limitations in being able to measure institutional changes and that some types of studies may not be conducive to randomisation, we are including a number of study designs that meet the inclusion criteria for the Cochrane Effective Practice and Organisation of Care (Cochrane Effective Practice and Organisation of Care, 2017).

These include:

- Randomised trials: An experimental study in which people are allocated to different interventions using methods that are random. Including head‐to‐head studies and studies with control groups not receiving the intervention. Participants may be assigned to interventions individually or by group (cluster‐randomised trials).
- Non‐randomised trial: An experimental study in which people are allocated to different interventions using methods that are not random. As per Cochrane Effective Practice and Organisation of Care recommendations, we will accept non‐randomised trials with at least two intervention sites and two control sites.
- Controlled before‐and‐after studies: A study in which observations are made before and after the implementation of an intervention, both in a group that receives the intervention and in a control group that does not. Allocation is usually determined by other factors outside the control of the investigators.

And quasi‐experimental designs including:

- Interrupted time series studies: A study that uses observations at multiple time points before and after an intervention (the ‘interruption’). The design attempts to detect whether the intervention has had an effect significantly greater than any underlying trend over time. Where an interrupted time series study includes measurements made in the same individuals at each time point, it is called a repeated measures study. As per Cochrane Effective Practice and Organisation of Care recommendations we will accept interrupted time series that include at least three data points before and three after the intervention. We will also exclude studies that do not have a clearly defined point in time when the intervention occurred.
- Regression discontinuity designs: A quasi‐experimental, pretest‐posttest control group design that is characterised by its unique method of assignment to intervention. Participants are assigned to either the intervention group or control group solely on the basis of a cut‐off score on a pretest measure. The design is so named because a regression line is plotted to relate the assignment and outcome variables. If the treatment is effective, a discontinuity in the regression line should occur at the cut‐off point. By comparison, the absence of a discontinuity is interpreted as a null effect.
- Difference of difference or other econometric designs: A quasi‐experimental design that makes use of longitudinal data from treatment and control groups to obtain an appropriate counterfactual to estimate a causal effect. Typically used to estimate the effect of a specific intervention or treatment (such as a passage of law, enactment of policy or large‐scale programme implementation) by comparing the changes in outcomes over time between a population that is enroled in a programme (the intervention group) and a population that is not (the control group).
- Propensity score matching and other matching designs: Propensity score matching creates sets of participants for treatment and control groups. A matched set consists of at least one participant in the treatment group and one in the control group with similar propensity scores. The technique attempts to estimate the effect of a treatment, policy or other intervention by accounting for the covariates that predict receiving the treatment.

The above implies that the following study designs will be excluded from this EGM:

- Non‐controlled pre‐post evaluations
- Case studies
- Cross‐sectional studies
- Observational studies
- Opinion pieces, editorials

#### 3.3.2 Treatment of qualitative research

We do not plan to include qualitative research.

#### 3.3.3 Types of settings

The EGM is global and will include studies conducted in low‐, middle‐ and high‐income countries.

The EGM will not be limited to at‐risk populations only but take a whole‐of‐population approach and thereby include universal, targeted and intensive interventions (i.e., primary, secondary and tertiary prevention approaches).

For this EGM, ‘institutional setting’ has been defined as an establishment or organisation used to promote child‐related activities and/or care. Based on this definition, studies conducted in the following settings will be eligible:

- Kindergarten / preschool / centre‐based early childhood education and care settings,
- Schools / before and after‐school care settings,
- Sports clubs, sport and recreation settings,
- Dance, drama and music studios / schools,
- Churches / religious institutions,
- Summer / vacation camps,
- Out‐of‐home care settings (OOHC) / foster care
- Orphanages
- Detention centres / juvenile justice settings
- Rescue centres
- Hospitals / health clinics / emergency departments^1^
- Any other type of organisation / institutional setting in which children may spend their time.

Studies written in the following languages will be included:

- English
- German
- French
- Spanish
- Italian
- Portuguese
- Dutch
- Danish
- Swedish
- Norwegian

No limitations will be put on the years in which studies were published.

#### 3.3.4 Status of studies

This EGM will include both finalised and on‐going studies. Ongoing studies will be identified through registered protocols based on database searches / trial registries and grey literature searches.

### 3.4 Search strategy and status of studies

This EGM will be based on the following search strategy:

#### 3.4.1 Academic databases

The following electronic databases will be searched for eligible studies (suggested search terms can be seen at Appendix A):

- Medline
- PsycInfo
- CINAHL
- ERIC
- Family and Society Studies Worldwide
- SocIndex
- Scopus
- The Campbell Collaboration Library

#### 3.4.2 Trial registries

- PROSPERO
- ClinicalTrials.gov (US)
- ISRCTN registry (UK)
- EU Clinical Trials Register
- Australia and New Zealand clinical trial registry (ANZCTR)

#### 3.4.3 Grey literature

Grey literature to be included in this EGM will be sourced based on the following sources:

**Table:** 

*Org. websites*	*Grey literature databases*	*Inquiries*
US Child Welfare Services	Dissertation repository ProQuest	Royal Commission into Institutional Responses to Child Sexual Abuse (Australia, 2014–2017)
World Health Organization		Pennsylvania Attorney General (2018). Pennsylvania Diocese Victims Report.
World Bank		MHG Studie—Sexueller Missbrauch an Minderjährigen durch Kleriker
UNICEF		Law Commission of Canada (2012). Restoring Dignity—Responding to Child Abuse in Canadian Institutions
Australian Institute for Family Studies		The Scottish Government (2012). Review of Child Neglect in Scotland
London School of Hygiene and Tropical Medicine		New Zealand House of Representatives (2014). Inquiry into Improving Child Health Outcomes and preventing child abuse with a focus from preconception until three years of age
National Institute for Health and Care Excellence		Northern Ireland Historical Institutional Abuse Inquiry 1922–1995 (2017)
National Society for the Prevention of Cruelty to Children		Unabhängige Kommission zur Aufarbeitung sexuellen Kindesmissbrauchs (Germany, 2016–2023)
Better Care Network		

The search for grey literature can be expanded based on input from multiple stakeholders (further described below).

Given the registration of multiple EGMs related to child maltreatment and violence against children, our research team will collaborate with the team behind the Pundir et al. (2019) EGM focused on violence against children in LMICs to exchange relevant grey literature potentially relevant to either of the two EGMs.

#### 3.4.4 Asking experts

The members of our Subject Matter Experts group (for a description see further below) will be invited to (a) forward studies of potential relevance to this EGM and (b) make their networks aware of the project and invite these to forward potentially relevant studies.

### 3.5 Screening and selection of studies

Two independent reviewers will screen all literature for inclusion at both title‐abstract and full‐text level. Conflicts will be resolved by a third member of the research team. Authors who are involved in primary studies will not be involved in the screening and selection of studies.

The Covidence platform will be used for literature screening. There will be no use of automation or text‐mining.

### 3.6 Data extraction, coding and management

Two coders will independently code all eligible studies / reviews. Conflicts will be resolved by a third member of the research team. Where information is unavailable from published reports, we will contact study authors to obtain such data. Multiple reports of the same study will be collated to ensure that each study rather than each report is the unit of interest in the review. Authors who are involved in primary studies will not be involved in the data extraction / coding / critical appraisal of those studies.

A preliminary version of the coding scheme to be used is available with Appendix B to this protocol. This scheme will be pretested with a selected sample of included studies / reviews representing the entire range of study designs eligible for this EGM. It will be refined and adjusted as required and then used with all studies / reviews included. Reviewers will initially extract data from 10 articles with responses assessed both against pre‐defined criteria and compared to each other. Inter‐reviewer agreement, consistency of comprehension and application will be assessed, and additional training initiated where necessary. Following, weekly ongoing spot checks will be completed on a random sample (at least 10% in total) of studies.

### 3.7 Quality appraisal

Only systematic reviews and RCTs will be assessed for quality using the following tools:

The AMSTAR‐2 tool for Systematic Reviews (Shea et al., 2017; Appendix C). Each systematic review will be independently rated by two reviewers. Conflicts will be resolved by a third member of the research team.

Each RCT will be assessed for quality using the Cochrane Risk of Bias 2 tool (Appendix D; Higgins et al., 2016). Risk of bias will be assessed independently by two reviewers, a third reviewer will adjudicate discrepancies regarding the risk of bias that cannot be resolved via consensus.

## 4 ANALYSIS AND PRESENTATION

### 4.1 Unit of analyses

Each entry in this EGM will either be an overview of systematic reviews, a systematic review or a primary impact evaluation.

The final EGM will identify the number of studies covered by the map in each sector or sub‐sector.

### 4.2 Planned analyses

The EGM will be supplemented by an EGM report that—based on tables and / or graphs—will descriptively summarise the number of studies included in the EGM and their distribution across different coding categories such as study type, geography, maltreatment type, target populations, interventions and outcomes. Each table / graph will be accompanied by brief narrative descriptions.

The report will also discuss the potential use of the EGM and highlight its boundaries and limitations.

### 4.3 Presentation

The anticipated presentation of the EGM was provided above as part of the methodology section.

## 5 STAKEHOLDER ENGAGEMENT

The scope of this EGM was developed in close collaboration between:

- Porticus, which is funding the study, represented by
  ○ Jane Leek, Regional Director, Porticus UK
  ○ Joachim Krapels, Senior Analyst, Porticus Effective Philanthropy Group

- Giving Evidence, represented by its CEO Caroline Fiennes.
- The Centre for Evidence and Implementation, represented by Directors Bianca Albers and Dr Robyn Mildon.
- Monash University, represented by Professor Aron Shlonsky.

This team will continue to collaborate around producing this EGM and facilitate joint decision‐making.

Furthermore, subject matter experts representing sixteen different organisations concerned with safeguarding practice and research will be convened for the production of this EGM to ensure that all relevant aspects of child maltreatment within institutional settings are sufficiently captured.

This group has been gathered once already to a general information and engagement meeting. In subsequent steps, it will be involved in sourcing relevant publications to be considered for the EGM and in disseminating the final EGM amongst relevant organisations, institutions and networks around the world. The current composition of this group is reflected in the table below. Further experts may be added as the EGM production progresses.

**Table:** 

	Name	Organisational affiliation	Country
**1**	Professor Leah Bromfield	Australian Centre for Child Protection, University of South Australia	AUS
**2**	Professor Daryl Higgins	Institute of Child Protection Studies, Australian Catholic University	AUS
**3**	Professor Ben Mathews	Director, Childhood Adversity Research Program, Faculty of Health, Queensland University of Technology	AUS
**4**	Emeritus Professor Stephen Smallbone	Griffith University, Australia	AUS
**5**	Mathieu Lacambre/Wayne Bodkin	Department of Forensic Psychiatry, University Hospital Montpellier	F
**6**	Dr Karen Devries/Louise Knight	London School of Hygiene and Tropical Medicine, UK	UK
**7**	Donald Findlater/Stuart Allardyce	‘Stop It Now’/Lucy Faithfull Foundation, UK	UK
**8**	Honorary Professor Derek E. Perkins	School of Law, Royal Holloway University of London, UK	UK
**9**	Professor Richard Wortley & Lorraine Sherr	University College London, UK	UK
**10**	Francisca Meinck	Oxford University	UK
**11**	Professor Elizabeth J. Letourneau	Johns Hopkins Bloomberg School of Public Health	US
**12**	Professor Jennie Noll	Penn State College of Health and Human Development	US
**13**	Dr Bruce Taylor	NORC, University of Chicago	US
**14**	Nicole Williams	Maestral International	INT
**15**	Kerry Albright	UNICEF	INT
**16**	Claire Feinstein	Save the Children	INT

## ROLES AND RESPONSIBILITIES

The three key competency areas—content, methods and information retrieval—required for this EGM will be covered by research team members in the following way:

- **Content**: Professor Aron Shlonsky and Dr Robyn Mildon will provide content expertise in producing this EGM. Joachim Krapels will provide content‐focused input to the development of the map, representing the perspective of the funder—Porticus (described further below)
  – ***Professor Aron Shlonsky*** is Professor and Head of Social Work at Monash University in Melbourne, Australia. After graduating from UC Berkeley with a doctorate in social welfare and a master's degree in public health, Shlonsky was an Assistant Professor at Columbia University School of Social Work and was then Factor‐Inwentash Chair in Child Welfare at the University of Toronto Faculty of Social Work. Shlonsky is known internationally for his work in risk assessment for child maltreatment and domestic violence, child welfare practice and policy, data analytics and the use of evidence to inform practice and policy. He also has had a long‐term involvement with the Campbell Collaboration. Shlonsky has been Investigator and Co‐Investigator on a large number of impact evaluations of systems that provide services to children and families and has authored and co‐authored numerous other books and peer‐reviewed articles on issues related to evidence‐based child welfare practice and child maltreatment. This includes the lead‐authorship for multiple reports commissioned by the Australian Royal Commission into Institutional Responses to Child Sexual Abuse between 2014 and 2017.
  – ***Dr Robyn Mildon*** is an internationally recognised figure in the field of evidence synthesis and translation, implementation science and programme and policy evaluations in health and human services. She is the Founding Executive Director of the Centre for Evidence and Implementation (CEI), an Honorary Associate Professor with the University of Melbourne, and the inaugural Co‐Chair of the Knowledge Translation and Implementation Group with the Campbell Collaboration, an international systematic review group. Over her career, Robyn has led a number of projects focused on the generation of evidence through systematic review methods and the better use of evidence in policy and practice through the study and application of implementation science. She has a substantial track record working with multiple stakeholders to support the adoption, implementation and evaluation of effective approaches to working with children, families and their communities, and advancing the use of evidence in practice
  – ***Dr Joachim Krapels*** is a Senior Analyst at Porticus, supporting the production of this EGM. He provided early input on the scope, framing and content of the EGM, bringing the perspective of Porticus to the work. He will not be engaged in detailing the methods for producing this EGM, the retrieval, screening and assessing of literature or the synthesis of findings. However, he will review findings and as part of that process provide suggestions from perspective of the funder.

- **EGM methods**: Meghan Finch, Professor Aron Shlonsky and Bianca Albers will provide methods expertise in producing this EGM
  – ***Meghan Finch*** Dr Finch has extensive experience in conducting systematic literature reviews for policy and practice use. She is a senior advisor specialising in synthesis with the Centre for Evidence and Implementation, a conjoint lecturer with the University of Newcastle, and Managing Editor of the Knowledge Translation and Implementation Group with the Campbell Collaboration, an international systematic review group. Over her career she has lead or co‐authored multiple systematic reviews including Cochrane reviews. She also has a substantial track record leading large scale trials focusing on supporting the adoption, implementation and evaluation of effective approaches to implementing evidence‐based policies and practices in the early childhood education and care sector.
  – ***Bianca Albers*** is a Director, heading CEI's work with knowledge synthesis and translation. Ms Albers has extensive experience in conducting different types of systematic literature reviews for policy and practice use. She has been involved in the production of full systematic reviews, rapid evidence assessments, scoping reviews, reviews of reviews and evidence and gap maps—commissioned among others by the VIC Department of Health and Human Services Australia (EGM on child and family services); the NSW Department of Family and Community Services (Australia – EGM on OOHC); World Health Organisation (full systematic reviews on Community Health Worker Programmes), and The Royal Commission into Institutional Responses to Child Sexual Abuse (multiple projects).
  – In questions that require particular methodical and/or statistical expertise, the team will be supported by Professor Aron Shlonsky, who also will review further key documents outlining the methods for this EGM before these are applied.

- **Information retrieval:** The production of this EGM will be supported by a Monash University librarian.
  In addition to the above functions, the daily management of the EGM production, including coordination of multiple, parallel work processes, stakeholder engagement and communication and other responsibilities will be shared by Caroline Fiennes and Meghan Finch.
  The screening of literature, quality assessments and coding of studies and comparable research tasks will be solved by two research assistants—Ludvig Bjørndal and James Hennessy—under the daily supervision of Meghan Finch. If issues—e.g., particular methodical questions—require specialist expertise, Professor Shlonsky will be involved on an ‘as‐needed’ basis.

## SOURCES OF SUPPORT

The proposed EGM will be funded by Porticus, an international organisation managing and developing the philanthropic programmes of charitable entities established by Brenninkmeijer family entrepreneurs. Porticus is involved with and fund a broad range of social service activities, including to both faith‐based organisations, and organisations unrelated to religious institutions.

## DECLARATIONS OF INTEREST

**Porticus** has supported, and currently supports, efforts among its grantees to improve organisational safeguarding of children. Specifically, Porticus

- requires from all its grantees to have a safeguarding policy
- works with some grantees to further develop interventions that can make the organisations safer.

These projects are conducted in collaboration with both faith‐based organisations and non‐faith‐based organisations. To ensure that all standards for the production of a Campbell EGM are met, Porticus has not been and will not be involved in any technical steps taken to produce the EGM, including information retrieval, data analysis and reporting of findings.

Porticus commissions this EGM to further support its own and others' ongoing work to enhance organisational safeguarding.

**Professor Aron Shlonsky, Dr Robyn Mildon and Ms Bianca Albers** have co‐authored multiple publications commissioned by the Australian Royal Commission into Institutional Responses to Child Sexual Abuse including the following:

- **Shlonsky, A., Albers, B.** & Paterson, N. (2017). Research Report ‐ Rapid evidence review on the availability, modality and effectiveness of psychosocial support services for child and adult victims and survivors of child sexual abuse ‐ Treatment and support needs (pp. 1–85). Melbourne: University of Melbourne ‐ School of Health Sciences.
- **Shlonsky, A., Albers, B.**, Tolliday, D., Wilson, S. J., Norvell, J. & Kissinger, L. (2017). Rapid evidence assessment: Current best evidence in the therapeutic treatment of children with problem or harmful sexual behaviours, and children who have sexually offended (pp. 1–114). Sydney: Royal Commission into Institutional Responses to Child Sexual Abuse.
- valentine, K., Katz, I., Smyth, C., Bent, C., Spada‐Rinaldis, S., Wade, C. & **Albers, B.** (2016). Key Elements of Child Safe Organisations – Research Study (pp. 1–112). Sydney: Royal Commission into Institutional Responses to Child Sexual Abuse, Commonwealth of Australia.
- **Albers, B.**, & **Mildon, R.** (2016). Implementation best practice: A rapid evidence assessment (pp. 1–143). Melbourne: The Parenting Research Centre for the Royal Commission into Institutional Responses to Child Sexual Abuse.
- South, S., **Shlonsky, A.** & **Mildon, R.** (2014). Scoping review: Evaluations of out‐of‐home care practice elements that aim to prevent child sexual abuse. Royal Commission into Institutional Responses to Child Sexual Abuse.
- South, S., **Shlonsky, A.** & **Mildon, R.** (2015). Scoping review: Evaluations of pre‐employment screening practices for child‐related work that aim to prevent child sexual abuse. Royal Commission into Institutional Responses to Child Sexual Abuse.

## PRELIMINARY TIMEFRAME

This EGM is expected to fully be available in August 2019.

## PLANS FOR UPDATING THE EGM

This EGM will be updated on a biennial basis.

## Footnotes

1 Note that medical interventions are excluded; however, of interest are: Interventions that aim to support health staff – e.g. GPs, nurses and doctors – to better identify / prevent maltreatment because these professions work in other institutional settings; for example: sport clubs have doctors appointed to work with their young talented soccer players; nurses may work with kids in residential care etc.

## APPENDIX A SUGGESTED SEARCH TERMINOLOGY WITH RESULTS

Database: psychinfo

Searched on April 3rd, 2019

**Table:**

#	Searches	Results
1	(Infant or infants or infancy or Child or childs or children or childrens or childhood or Minors or Minor person* or minor people or Toddler or toddlers or baby or babies or Adolescent or adolescents or adolescence or teen or teens or teenage or teenaged or teenager or teenagers or young person or young persons or young people or youth or youths or juvenile or juveniles or boy or boys or girl or girls).mp. or (adolescence 13 17 year or childhood birth 12 year or infancy 2 23 month or neonatal birth 1 month or preschool age 2 5 year or school age 6 12 year).ag.	1151042
2	(Infant or infants or infancy or Child or childs or children or childrens or childhood or Minors or Minor person* or minor people or Toddler or toddlers or baby or babies or Adolescent or adolescents or adolescence or teen or teens or teenage or teenaged or teenager or teenagers or young person or young persons or young people or youth or youths or juvenile or juveniles or boy or boys or girl or girls).mp.	969837
3	((neglect* or abandon* or maltreat* or mistreat* or ill treat* or illtreat* or harm or harmful or harmed or vulnerab* or abus* or assault) or (harmful sexual behavi* or problem sexual behavi*)).mp	309336
4	1 and 3	123067
5	2 adj3 3	54474
6	4 or 5	123067
7	(metaanal* or meta anal* or (systematic adj2 review*) or systematic synthesis).mp. or (meta analysis or metasynthesis or "systematic review").md	57578
8	(RCT or randomi* or (random* adj3 (assign* or allocat*)) or blinded or double blind* or doubleblind*).mp	123101
9	(Quasi experiment* or quasiexperiment* or step wedge or difference in difference* or synthetic control group or covariate matching or propensity score or doubly robust estimat* or regression adjustment estimate* or regression discontinuity or instrumental variable* estimate* or time series or timeseries or before after or before‐after or pre post).mp	29644
10	or/7–9	197943
11	(intervention or interventions or prevent* or treatment or treatments or program or programs or programme or programmes or policy or policies).mp	1340817
12	Health Education/ or Mass Media/ or Prevention/ or Social Media/ or Communications Media/	60371
13	professional development/ or continuing education/ or inservice teacher education/ or inservice training/ or training/ or professional training/ or mental health inservice training/ or professional certification/ or professional competence/ or professional standards/	50160
14	(Human Resource Management or Job Applicant Screening or Personnel Recruitment or employ* screening or pre employ* screening).mp	14872
15	Or/11–14	1396199
16	((residential and (care or institution)) or (oohc or (out of home adj3 care*)) or (foster* adj2 (youth or child* or infant*)) or (child* adj2 looked after) or orphanage or (child* adj2 home) or (child* adj2 institution) or pre school or preschool or pre k or kindergarten or day care or daycare or nursery or nurseries or play group* or playgroup* or ((after school or afterschool or out of school) and program*) or camp or camps or club or clubs or (child* and (center* or centre* or institution*)) or (institution* adj2 (faith based religious or care or setting)) pr church* or temple* or mosque*).mp	172124
17	exp Correctional Institutions	9423
18	junior high schools/ or technical schools/ or middle schools/ or nursery schools/ or elementary schools/ or nongraded schools/ or military schools/ or high schools/ or charter schools/ or boarding schools/ or schools/ or institutional schools/	48033
19	Or/16–18	224041
20	6 and 10 and 15 and 19	688

## APPENDIX B EGM CODING SCHEME

***Study charcteristics***

1. **Study design**
  1.1. Systematic review
  1.2. RCT (including cluster RCT)
  1.3. QED
  1.4. Unclear
2. **Status of study**
  2.1. Completed
  2.2. Ongoing
  2.3. Unclear
3. **Systematic review quality**
  3.1. Low (AMSTAR score 0–3)
  3.2. Moderate (AMSTAR score 4–7)
  3.3. High (AMSTAR score 8–11)
4. **Primary study quality**
  4.1. Low
  4.2. Moderate
  4.3. High

***Population***

5. **Target population**
  5.1. Child victims
  5.2. Child offenders
  5.3. Institutional adult members
  5.4. Adult perpetrators
  5.5. Mixed
  5.6. Unclear
6. **Child victim/offender age group(s)**
  6.1. Prenatal
  6.2. Infancy (0–23 months)
  6.3.Early childhood (24 months –5 years)
  6.4. Middle childhood (6–11 years)
  6.5. Early adolescence (12–14)
  6.6. Late adolescence (15–17)
  6.7. Mixed
  6.8. Unclear
7. **Child risk status**
  7.1. Not at risk population
  7.2. At risk population
  7.3. Exposed population
  7.4. Mixed population
  7.5. Unclear
8. **Type of maltreatment**
  8.1. Neglect
  8.2. Physical abuse
  8.3. Sexual abuse
  8.4. Emotional abuse
  8.5. Mixed
  8.6. Unclear

***Intervention***

9. **Intervention type**
  9.1. Prevention
  9.2. Disclosure
  9.3. Response
  9.4. Treatment
  9.5. Other: _________________
  9.6. Mixed
  9.7. Unclear
10. **Intervention target**
  10.1. Child victim
  10.2. Child offender
  10.3. Adult perpetrator
  10.4. Organisational leadership
  10.5. Organisational staff
  10.6. Caregiver / parent
  10.7. Other: _________________
  10.8. Mixed
  10.9. Unclear
11. **Delivery mode**
  11.1 Individual
  11.2. Group
  11.3 Other: ______________________
  11.4. Mixed
  11.5. Unclear

***Setting***

12. **Geography (following WHO Regions)**
  12.1. Africa
  12.2. Americas
  12.3. South‐East Asia
  12.4. Europe
  13.5. Eastern Mediterranean
  12.6. Western Pacific
13. **Institutional Setting**
  13.1. Day care
  13.2. School (including preschool/after‐school)
  13.3. Sports club
  13.4. Churches/religious institutions
  13.5. Summer/vacation camps
  13.6. Residential care (including orphanages)
  13.7. Detention centres/juvenile justice settings
  13.8. Rescue centres
  13.9. Primary health care facilities
  13.10. Secondary health care facilities
  13.11. Other: ___________________
  13.12. Mixed
  13.13. Unclear

***Outcomes***

14. **Institutional practice**
  14.1. Institutional culture
  14.2. Operational practice
  14.3. Environmental
15. **Disclosure**
  15.1. Disclosure rates
16. **Child maltreatment**
  16.1. Maltreatment occurrence
17. **Child cognitive functioning**
  17.1. Academic achievement
  17.2. School engagement
  17.3. Problem solving skills
  17.4. Decision making skills
18. **Child physical health and development**
  18.1. Overall health
  18.2. BMI
  18.3. Health related risk‐avoidance behaviour
19. **Child mental health**
  19.1. Internalising symptoms
  19.2. Externalising symptoms
  19.3. Traumatic stress symptoms
  19.4. Coping
  19.5. Resilience
  19.6. Quality of life/subjective wellbeing
20. **Child social functioning**
  20.1. Social competence
  20.2. Social skills
  20.3. Adaptive behaviours
  20.4. Social relationships and network
  20.5. Family situation
21. **Child offender outcomes**
  21.1. Behaviour change
  21.2. Relapse into maltreatment
  21.3. Other: ______________________
22. **Adult perpetrator outcomes**
  22.1. Recidivism
  22.2. Desistance
  22.3. Other: ______________________
23. **Parent/caregiver outcomes**
  23.1. Knowledge/Awareness
  23.2. Other: ______________________

## APPENDIX C AMSTAR‐2 TOOL

**Table:**

## APPENDIX D COCHRANE RISK OF BIAS 2 TOOL

Domain 1: Risk of bias arising from the randomisation process

**Table:**

**Table:**

Signalling questions		Response options
**1.1 Was the allocation sequence random?**		
**1.2 Was the allocation sequence concealed until participants were enrolled and assigned to interventions?**		
**1.3 Did baseline differences between intervention groups suggest a problem with the randomization process?**		
**Risk‐of‐bias judgement**		Low/High/Some concerns
Optional: What is the predicted direction of bias arising from the randomization process?		Favours experimental/Favours comparator/Towards null/Away from null/Unpredictable

Domain 2: Risk of bias due to deviations from the intended interventions (*effect of assignment to intervention*)

**Table:**

Signalling questions		Response options
**2.1. Were participants aware of their assigned intervention during the trial?**		
**2.2. Were carers and people delivering the interventions aware of participants' assigned intervention during the trial?**		
**2.3. *If Y/PY/NI to 2.1 or 2.2*: Were there deviations from the intended intervention that arose because of the experimental context?**		
**2.4. *If Y/PY to 2.3*: Were these deviations from intended intervention balanced between groups?**		
**2.5 *If N/PN/NI to 2.4*: Were these deviations likely to have affected the outcome?**		
**2.6 Was an appropriate analysis used to estimate the effect of assignment to intervention?**		
**2.7 *If N/PN/NI to 2.6* : Was there potential for a substantial impact (on the result) of the failure to analyse participants in the group to which they were randomized?**		
**Risk‐of‐bias judgement**		Low/High/Some concerns
Optional: What is the predicted direction of bias due to deviations from intended interventions?		Favours experimental/Favours comparator/Towards null/Away from null/Unpredictable

Domain 2: Risk of bias due to deviations from the intended interventions (*effect of adhering to intervention*)

**Table:**

Signalling questions		Response options
**2.1. Were participants aware of their assigned intervention during the trial?**		
**2.2. Were carers and people delivering the interventions aware of participants' assigned intervention during the trial?**		
**2.3. *If Y/PY/NI to 2.1 or 2.2*: Were important co‐interventions balanced across intervention groups?**		
**2.4. Could failures in implementing the intervention have affected the outcome?**		
**2.5. Did study participants adhere to the assigned intervention regimen?**		
**2.6. *If N/PN/NI to 2.3 or 2.5 or Y/PY/NI to 2.4*: Was an appropriate analysis used to estimate the effect of adhering to the intervention?**		
**Risk‐of‐bias judgement**		Low/High/Some concerns
Optional: What is the predicted direction of bias due to deviations from intended interventions?		Favours experimental/Favours comparator/Towards null /Away from null / Unpredictable

Domain 3: Missing outcome data

**Table:**

Signalling questions		Response options
**3.1 Were data for this outcome available for all, or nearly all, participants randomized?**		
**3.2 *If N/PN/NI to 3.1*: Is there evidence that result was not biased by missing outcome data?**		
**3.3 *If N/PN to 3.2*: Could missingness in the outcome depend on its true value?**		
**3.4 *If Y/PY/NI to 3.3*: Do the proportions of missing outcome data differ between intervention groups?**		
**3.5 *If Y/PY/NI to 3.3*: Is it likely that missingness in the outcome depended on its true value?**		
**Risk‐of‐bias judgement**		Low/High/Some concerns
Optional: What is the predicted direction of bias due to missing outcome data?		Favours experimental / Favours comparator/Towards null /Away from null/Unpredictable

Domain 4: Risk of bias in measurement of the outcome

**Table:**

Signalling questions		Response options
**4.1 Was the method of measuring the outcome inappropriate?**		
**4.2 Could measurement or ascertainment of the outcome have differed between intervention groups?**		
**4.3 If N/PN/NI to 4.1 and 4.2: Were outcome assessors aware of the intervention received by study participants?**		
**4.4 If Y/PY/NI to 4.3: Could assessment of the outcome have been influenced by knowledge of intervention received?**		
**4.5 If Y/PY/NI to 4.4: Is it likely that assessment of the outcome was influenced by knowledge of intervention received?**		
**Risk‐of‐bias judgement**		Low/High/Some concerns
Optional: What is the predicted direction of bias in measurement of the outcome?		Favours experimental/Favours comparator/Towards null/Away from null/Unpredictable

Domain 5: Risk of bias in selection of the reported result

**Table:**

Signalling questions		Response options
**5.1 Was the trial analysed in accordance with a pre‐specified plan that was finalized before unblinded outcome data were available for analysis?**		
**Is the numerical result being assessed likely to have been selected, on the basis of the results, from…**		
**5.2 … multiple outcome measurements (e.g., scales, definitions, time points) within the outcome domain?**		
**5.3 … multiple analyses of the data?**		
**Risk‐of‐bias judgement**		Low/High/Some concerns
Optional: What is the predicted direction of bias due to selection of the reported result?		Favours experimental/Favours comparator/Towards null/Away from null/Unpredictable

Overall risk of bias

**Table:**

**Risk‐of‐bias judgement**		Low/High/Some concerns
Optional: What is the predicted direction of bias due to selection of the reported result?		Favours experimental/Favours comparator/Towards null/Away from null/Unpredictable
